# Role of Peritoneal Mesothelial Cells in the Progression of Peritoneal Metastases

**DOI:** 10.3390/cancers14122856

**Published:** 2022-06-09

**Authors:** Junliang Li, Tiankang Guo

**Affiliations:** 1 The First School of Clinical Medicine, Lanzhou University, Lanzhou 730030, China; lijl2018@lzu.edu.cn; 2 Department of General Surgery, Gansu Provincial Hospital, Lanzhou 730030, China; 3 The First School of Clinical Medical, Gansu University of Chinese Medicine, Lanzhou 730030, China

**Keywords:** peritoneal mesothelial cells, peritoneal metastatic cancer, mesothelial–mesenchymal transition

## Abstract

**Simple Summary:**

Peritoneal mesothelial cells (PMCs) are the first cell layer to encounter peritoneal tumors, thereby affecting their progression. In the past, PMCs were considered a barrier to the intraperitoneal implantation and metastasis of tumors. However, the specific role of PMCs in local tumor progression remains controversial, with evidence demonstrating both inhibiting or promoting effects. The epithelial phenotype of PMCs exhibits an inhibitory effect on abdominal cavity metastasis, whereas the mesenchymal phenotype of PMCs significantly promotes the progression of abdominal cavity tumors. Nevertheless, the details of the interaction between PMCs and tumors to promote local progression and peritoneal dissemination require further study. This article presents a review of the role of PMCs in the progression of abdominal tumors. Based on existing evidence, maintaining the stability of the PMC epithelial phenotype may be a valuable target for inhibiting peritoneal tumor dissemination and local progression.

**Abstract:**

Peritoneal metastatic cancer comprises a heterogeneous group of primary tumors that originate in the peritoneal cavity or metastasize into the peritoneal cavity from a different origin. Metastasis is a characteristic of end-stage disease, often indicative of a poor prognosis with limited treatment options. Peritoneal mesothelial cells (PMCs) are a thin layer of cells present on the surface of the peritoneum. They display differentiated characteristics in embryonic development and adults, representing the first cell layer encountering peritoneal tumors to affect their progression. PMCs have been traditionally considered a barrier to the intraperitoneal implantation and metastasis of tumors; however, recent studies indicate that PMCs can either inhibit or actively promote tumor progression through distinct mechanisms. This article presents a review of the role of PMCs in the progression of peritoneum implanted tumors, offering new ideas for therapeutic targets and related research.

## 1. Introduction

Peritoneal metastases comprise a heterogeneous group of primary tumors that originate in the peritoneal cavity, such as ovarian cancer, gastric cancer, colorectal cancer, and pancreatic cancer, or tumors that originate outside the peritoneal cavity and metastasize into the peritoneal cavity, such as breast cancer, lung cancer, and melanoma. Peritoneal mesothelial cells (PMCs) cover the surface of the peritoneal cavity and are the first line of cells in contact with aggressive tumors. Thus, PMCs play a central role in the progression of peritoneal tumors. However, recent studies demonstrate both inhibitory and promoting effects of PMCs in local tumor progression and metastasis. Here, we provide an overview of the evidence of the role and mechanisms of PMCs in the progression of peritoneum implanted tumors.

## 2. Anatomical, Physiological, and Pathological Features of PMCs

### 2.1. Anatomy

The peritoneal cavity is the largest and most complex of the three serous cavities in the human body [1]. The visceral peritoneum covers the internal organs and the mesenteric surface. The parietal peritoneum covers the abdominal wall and the inner surface of the pelvic cavity, which connects with the visceral peritoneum to form the peritoneal cavity. The peritoneum consists of superficial PMCs, an underlying basement membrane, and a sub-peritoneal stroma [2]. PMCs are a monolayer of cells covering the peritoneal surface, originating from the mesoderm, and exhibit many characteristics of epithelial cells, including a polygonal cell shape, expression of surface microvilli, epithelial cytokeratins, and tight junctions between cells [3,4]. PMCs can simultaneously express the epithelial marker cytokeratins and the mesothelial markers vimentin and desmin. They can also gradually transition to a mesenchymal phenotype and lose their epithelial phenotype when triggered by changes in the microenvironment, a phenomenon known as the mesothelial–mesenchymal transition (MMT) [5,6].

### 2.2. Physiology

PMCs and their surface microvilli provide a smooth, non-adhesive surface for the peritoneal cavity. Under physiological conditions, the peritoneal cavity contains 5–20 mL of fluid that separates the two layers of the peritoneum. This plays a role in lubricating and protecting the organs [7]. The mesothelium is an essential part of the peritoneal barrier for water and ion transport [8]. The peritoneum is a semi-permeable membrane that allows the passive transport of fluids and solutes through intercellular junctions, hydrostatic and osmotic pressure gradients via the stomata, and active transport via the formation of (micro)pinocytotic vesicles [9,10,11]. The ion channels at the PMC membrane regulate the volume of PMCs according to a change in osmotic pressure [8,12,13,14,15,16].

### 2.3. Embryology

The peritoneum originates from the mesoderm and begins to develop during gastrulation. During gastrulation, PMCs are involved in embryonic development, which can migrate and transdifferentiate into endothelial cells, vascular smooth muscle cells, and other components [17,18]. Adult PMCs can differentiate into endoderm- and mesoderm-derived cells, and mature PMCs can differentiate into hepatic stellate cells (HSCs) [19], myofibroblasts, and macrophage-like cells [20]. There is increasing evidence that PMCs do not appear to be terminally differentiated and can further differentiate under specific physiological and pathological conditions [3].

### 2.4. Pathology

PMCs play important roles in pathological conditions, such as peritoneal dialysis dysfunction [21], intra-abdominal adhesions [22], peritoneal inflammation, peritoneal tumor dissemination [23], and endometriosis [24]. PMCs also play key roles in dynamic regulation processes such as peritoneal repair [25], scarring [26], the regulation of macrophage migration [27], and the promotion of coagulation [25,28], including the formation of fibrin and the promotion of fibrinolysis [29,30]. 

Peritoneal injury caused by long-term peritoneal dialysis, surgery, infection, or ischemia triggers a complex peritoneal defense response [2]. PMCs can promote the occurrence of peritoneal fibrosis through MMT, transforming growth factor-beta (TGF-β), and hypoxia-inducible factors, which are only some of the critical pathways involved in this process. The MMT of PMCs is also heavily involved in developing pathologies such as peritoneal metastasis [31] and endometriosis [24].

## 3. PMCs Inhibit Tumor Progression in the Peritoneal Cavity

Increasing evidence indicates that PMCs have tumor-inhibition ability, and act as the first barrier to gastrointestinal tumor dissemination in the peritoneal cavity. When SW-480 colon cancer cells or PSN-1 pancreatic cancer cells were injected into the abdominal cavity of nude mice, tumor aggressive behavior was significantly inhibited when primary human peritoneal mesothelial cells (HPMCs) derived from the human omentum were co-injected compared to that of tumor cells injected into the peritoneal cavity alone. Additional exploration demonstrated that HPMCs induced gastrointestinal tumor apoptosis by interacting with soluble sICAM-1 and CD43 [32].

Moreover, experiments with three-dimensional culture systems revealed that PMCs display a significant inhibitory effect on the adhesion and growth of ovarian cancer cell spheroids cultured in vitro. The culture supernatant of PMCs also showed a significant inhibitory effect on the proliferation of ovarian cancer cells [33]. An in vitro real-time imaging model demonstrated that ovarian cancer cell spheroids could infiltrate the sub-mesothelium by pushing apart PMCs via integrin- and talin-dependent myosin activation [34].

Further evidence supporting the inhibition of tumor dissemination by PMCs was obtained based on the immunohistochemical examination of clinical specimens, demonstrating the lack of PMCs below the metastatic lesion of the peritoneal dissemination of ovarian cancer, suggesting that PMCs may be the first barrier to ovarian cancer dissemination [35]. Experiments of tumorigenesis in nude mice and morphological investigation revealed that PMCs have the ability to inhibit tumor cells. Paradoxically, however, there is also gradually increasing evidence indicating that PMCs promote the progression and dissemination of peritoneal metastases.

## 4. Aging PMCs Promote Tumor Progression in the Peritoneal Cavity

Cellular senescence has been recognized as an effective tumor-suppressor mechanism that prevents cells at risk of malignant transformation from continuing to develop along the malignant course [36,37,38,39]. Recent studies have shown that senescent cells change their microenvironment through the secretion of relevant factors, and the senescence-associated secretory phenotype (SASP) of senescent cells is considered to potentially promote tumor progression [39]. 

**Figure 1:**
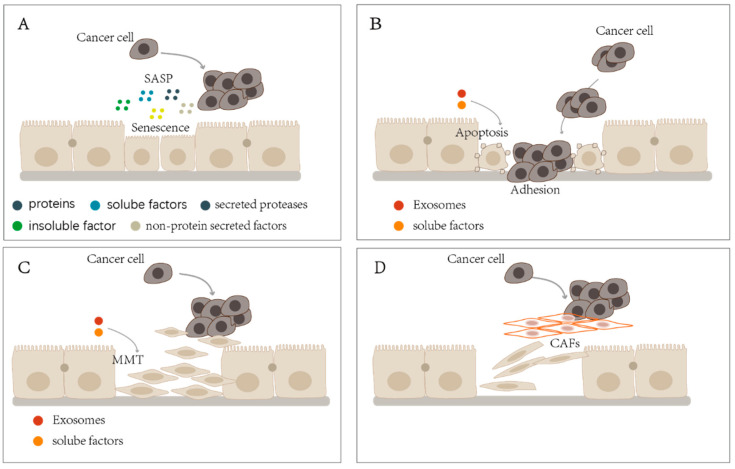
(**A**) Peritoneal mesothelial cells (PMCs) exhibit a senescence-associated secretory phenotype (SASP) after aging, which can secrete proteins, soluble factors, insoluble factors, secreted proteases, and non-protein factors to affect the tumor microenvironment and promote tumor progression. (**B**) PMCs undergo apoptosis and expose the sub-mesothelial tissue under the stimulation of tumor exosomes or soluble factors to promote tumor adhesion and progression. (**C**) PMCs undergo a mesothelial–mesenchymal transition (MMT) under the influence of tumor exosomes or soluble factors, which move in opposite directions from tumors and promote tumor progression. (**D**) PMCs promote tumor progression after transdifferentiation into cancer-associated fibroblasts (CAFs).

There are many substances secreted by senescent cells. First, these cells secrete proteins such as interleukin (IL)-1, IL-6, and IL-8 [40], which can affect surrounding cells by cell surface receptors, chemokines such as CXCL family-1/2/4/5/6/8/12, MCP-2/4, CCL family members 2/3/4/5/7/8/13/16/20/26, and CXCR-2 binding factor [41,42,43,44,45,46,47,48], and insulin-like growth factor (IGF) pathway components (IGFBP-2/3/4/5/6 and their regulators IGFBP-rP1/rP2/7), which influence the microenvironment of senescent cells [49,50,51,52,53]. Second, senescent cells secrete soluble factors such as colony-stimulating factors (CSFs), including granulocyte macrophage-CSF and G-CSF, osteoprotegerin, prostaglandin E2 (PGE2), and COX-2, which is the enzyme responsible for the production of PGE2 and other prostaglandins [46,50,54]. The third category includes secreted proteases such as matrix metalloproteinases (MMPs)-1/3/10, members of the plasminogen activation pathway family, including urokinase- or tissue-type plasminogen activators, and serine protease inhibitors, including plasminogen activator inhibitor (PAI)-1 and PAI-2 [46,54,55,56,57,58,59]. The fourth category is insoluble factors such as fibronectin, which is a prominent senescent marker that affects cell adhesion, survival, growth, and migration [60]. Finally, senescent cells also secrete non-protein factors, including nitric oxide and reactive oxygen species, which are known as modulators of the cellular phenotype [54,61,62] (Figure 1A).

Several studies have validated the tumor-progressing properties of aging PMCs. For example, gastric cancer cells can silence the endoglin expression in PMCs via the TGF-β pathway, thereby inducing the senescence of PMCs and thus enhancing the adhesion and invasion of gastric cancer cells [63]. In addition, a resveratrol derivative, 3,3′,4,4′,5,5′-hexahydroxy-trans-stilbene, could promote the proliferation and metastasis of colorectal and pancreatic cancer cells by inducing the senescence of PMCs in vitro [64].

Promotion of tumor progression by senescent primary PMCs derived from omentum cultures has also been observed in ovarian cancer. PMCs exhibited decreased proliferation and increased senescence when exposed to the ovarian cancer cell lines A2780, OVCAR-3, and SKOV-3, or malignant ascites from patients, and significantly promoted ovarian cancer cell adhesion, proliferation, and invasion [65]. Senescent PMCs promote the adhesion and proliferation of ovarian cancer cells in vitro by releasing CXCL1, CXCL8, IL-6, fibronectin (FN), and TGF-β1. In addition, senescent PMCs have also been detected in human ovarian cancer specimens [66]. Oxidative stress induces aging PMCs to upregulate FN expression, and the interaction of α5β1 integrin with HPMC-associated FN subsequently promotes the adhesion and invasion of ovarian cancer cells [67]. 

A significant characteristic of senescent cells is their reduced growth capacity. However, a thickened PMC layer has been observed near the peritoneal metastasis lesions of both colorectal and pancreatic cancers [68,69]. In addition, the proliferative and invasive ability of PMCs was found to be significantly enhanced when co-cultured with the NUGC4 gastric cancer cell line [70]. None of these studies supported the perspective that senescent PMCs promote tumor progression. Alternatively, the evidence above suggests that senescent PMCs may play a significant role in the promotion of tumor dissemination only during certain periods or is closely related to a specific tumor pathological subtype rather than being a widespread phenomenon.

## 5. Apoptotic PMCs Promote Tumor Progression in the Peritoneal Cavity

Paget’s “tumor and seeds” theory proposes that tumor cell metastasis is related to the tumor itself and to the microenvironment in which metastasis is likely to occur [71]. Confocal laser-scanning microscope observations confirmed that Fas (a 48 kDa membrane protein of cell surface death receptor) expressed on the membrane of PMCs and its ligand (FasL) expressed on the tumor cell membrane can interact. The colon cancer cell line SW480 can induce the apoptosis of PMCs via the FasL/Fas pathway, exposing the sub-mesothelial tissue for tumor adhesion [72].

The TGF-β1 signaling pathway plays a vital role in cell cycle regulation, growth and development, differentiation, extracellular matrix synthesis, and the immune response [73]. Many tumor-suppressor genes act through the TGF-β1 pathway [74,75]. TGF-β1 has a bidirectional regulatory effect on tumor progression, which can induce apoptosis and inhibit cell proliferation, thereby inhibiting tumor progression [73]. High expression of TGF-β1 has been associated with later clinical stages and worse prognosis in gastrointestinal tumors, including gastric, pancreatic, and colorectal cancers [76]. Co-incubation of gastric cancer cells and PMCs showed that the TGF-β1 secreted by gastric cancer cells could induce the apoptosis of PMCs while promoting the proliferation and metastasis of gastric cancer cells. The same phenomenon was also observed with the addition of TGF-β1 to the PMCs-only culture medium [77,78].

Recent studies have shown that tumor cells induce the apoptosis of PMCs via the secretion of soluble cytokines or extracellular vesicles before they arrive at the peritoneal implantation site for further metastasis (Figure 1B). Extracellular vesicles are rich in proteins, mRNAs, and microRNAs, which act as carriers for mediating intercellular communication. This can create a microenvironment conducive to metastasis prior to tumor cell arrival [79,80,81]. Ovarian cancer-derived extracellular vesicles deliver *MMP1* mRNA to PMCs to induce apoptosis and the consequent dissemination of ovarian tumors [82]. Gastric cancer-derived exosomes can destroy the physical barrier formed by PMCs, promote the apoptosis of PMCs, and enhance the adhesion and metastasis of gastric tumors [83]. MiR-106a from gastric cancer-derived exosomes can promote the apoptosis and MMT of PMCs by inhibiting the expression of SMAD7 [84]. Macrophage transformation to an M2 phenotype has been observed after co-culturing gastric cancer cells and macrophages. Following the second round of co-culture, PMCs underwent significant early and late apoptosis, accompanied by evident fibrosis. These changes are conducive to the peritoneum surface metastasis of gastric cancer cells [85]. PMCs can also have an inverse effect on tumor cells, promoting the peritoneal dissemination of colon cancer cells by releasing CD44-rich extracellular vesicles [86]. The apoptosis of PMCs can expose the sub-peritoneal connective tissue to encourage tumor adhesion; however, the details regarding tumor cell function enhancement require further study.

## 6. MMT of PMCs Promotes Tumor Progression in the Peritoneal Cavity

MMT in PMCs was first discovered in patients with chronic renal failure on peritoneal dialysis. PMCs can transition from an epithelial to a mesenchymal phenotype, characterized by the loss of epithelial morphology and epithelial markers, including cytokeratins and E-cadherin. The morphology of PMCs becomes phenotypically mesenchymal, accompanied by the development of migratory ability and increased expression of α-integrin [87]. The MMT of PMCs has also been associated with gastric, colorectal, ovarian, and other peritoneal metastatic cancers. Following MMT, PMCs acquire more robust, metastatic, and invasive features and properties to enhance tumor invasion [83,86,88]. 

Several mechanisms have been demonstrated by which PMCs acquire the MMT phenotype in gastrointestinal and gynecological tumors. PMCs can transition to the MMT phenotype under co-culture with cancer cells. For example, co-culturing of the highly metastatic MKN45 cell line and the PMC line HMrSV5 induced the MMT phenotype in PMCs, and the mesenchymal PMCs could in turn enhance the invasiveness and metastasis of the gastric cancer cells [70]. Furthermore, PMCs can be affected by soluble factors from the cancer environment. Connective tissue growth factor induces MMT in PMCs in a dose- and time-dependent manner, promoting the adhesion of gastric cancer cells to the peritoneum [89]. Moreover, cancer-derived exosomes can induce MMT in PMCs. After adding purified exosomes from the gastric cancer cell culture supernatant to HMrSV5 cells or administering the exosomes intraperitoneally to nude mice, the PMCs underwent MMT and promoted the peritoneal dissemination of gastric cancer [83]. Nicotinamide N-methyltransferase-containing exosomes derived from gastric cancer cells were also reported to induce MMT in PMCs and could promote the peritoneal metastasis of cancer cells through TGF-β/SMAD2 signaling [90]. Gastric cancer exosome-derived miR-21-5p induces MMT in PMCs and promotes tumor peritoneal dissemination by targeting SMAD7 [91]. A similar phenomenon was observed in ovarian cancer [92]. Purified exosomes from the supernatants of the OVCAR3 and ES-2 ovarian cancer cell lines are rich in ANXA2. The addition of HMrSV5 promoted the occurrence of MMT in PMCs via the PI3K/AKT/mTOR signaling pathway, and promoted the intraperitoneal implantation and metastasis of ovarian cancer cells [88]. Additionally, circPUM1 and circWHSC1 in ovarian cancer cells can be transferred to PMCs via exosomes, followed by inducing MMT to promote tumor metastasis [93,94].

MMT often presents prior to intraperitoneal metastasis (Figure 1C). Exosomes secreted by gastric cancer cells can be delivered and endocytosed to peritoneal PMCs by the activity of macrophages, which release exosome components to promote the occurrence of MMT in PMCs and prepare for peritoneal surface dissemination [95]. More than 70% of the IL-17A in the disseminated peritoneal tissues of gastric cancer was present in the mast cells, and the number of cells with double-positive immunostaining (IL-17A and mast cells) was positively correlated with peritoneal fibrosis. Moreover, the IL-17A released by mast cells was found to promote the peritoneal dissemination of gastric cancer by inducing the occurrence of MMT in PMCs in nude mice [96]. In addition, the interaction between PMCs and colon cancer was validated [86]. Interestingly, PMCs not only promoted tumor seeding and secondary invasion in the peritoneal cavity, but also played an active role in the local progression of gastric cancer, limited to the gastric wall. In another study [97], gastric cancer cells were injected into the gastric wall submucosa of nude mice, demonstrating that PMCs underwent MMT long before the gastric cancer cells locally infiltrated the serosa, and the PMCs also moved in the opposite direction to the gastric cancer cells, thus displaying apparent invasive ability. Moreover, a clear niche structure appeared on the peritoneal surface, preparing for the local infiltration of gastric cancer cells. The same phenomenon was seen in human gastric cancer specimens [97]. However, changes in PMC morphology are not limited to MMT and are often accompanied by changes in other biological processes. Primary PMCs from the human omentum developed aging caused by passing generations and displayed MMT features. Further exploration revealed that the response of aged PMCs to MMT after TGF-β stimulation is relatively weak. The above phenomenon may support the concept of cellular senescence being antagonistically pleiotropic regarding MMT in PMCs. To be sure, there is a relative link between aging and MMT in PMCs [98]. 

Peritoneal metastasis is the leading cause of treatment failure in gastric cancer, accounting for approximately 53–66% of distant metastases [99]. The current widely accepted view is that with the local progression of gastric cancer, gastric cancer cells will fall off into the peritoneal cavity after invading the serosa. Therefore, abdominal metastasis is considered a continuous pathological process, with the tumor only falling off into the peritoneum after invading the serosa [100]. It is perhaps confusing that some T2-stage and even T1-stage early gastric cancers also exhibit a positive preoperative peritoneal lavage cytology (CY1). Endo et al. [101] analyzed the clinicopathological data of 80 patients with CY1 gastric cancer, including 1, 1, 4, 42, and 9 patients in stages pT1, T2, T3, T4a, and T4b, respectively, 7.5% (6/80) of whom did not display penetration of the gastric serosa. Li et al. [102] studied 51 gastric cancer patients with preoperative laparoscopic exploration, and reported that 13.7% (7/51) of patients with stage cT3 cancer and below developed CY1 gastric cancer. Other gastric lavage studies have shown similar results [103,104,105,106,107].

Furthermore, this phenomenon is not limited to the local progression of gastric cancer. Preoperative peritoneal lavage has also been detected in 2% of patients with stage III colorectal cancer [108]. In an animal experiment, gastric cancer cells were injected into the abdominal cavity of nude mice. In the early stages of cancer cell invasion, PMCs were observed to undergo MMT and invade the muscle layer of the abdominal wall. Many PMCs were also found in the metastatic nodules in the abdominal cavity. These PMCs underwent MMT and expressed the mesenchymal marker alpha-smooth muscle actin (α-SMA) [97]. These results indicate that PMCs display prominent invasive characteristics after MMT to actively promote gastric cancer progression.

At present, there are two main approaches regarding D2 radical resection for gastric cancer: complete resection of the stomach or complete resection of the perigastric mesangium (also known as complete mesogastric excision). The latter involves dissection reaching the root of the artery, where the second lymph node around the stomach is located. A D2 operation is indicated in cases of tumor growth within the peritoneum surrounding the stomach. If the preoperative assessment indicates that the tumor has penetrated the peritoneum, perioperative treatment is usually recommended first, followed by surgery once the tumor retracts to the area surrounded by the peritoneum [109]. There are numerous cases of free cancer cells in the peritoneal cavity in clinical practice. However, the primary cancer is usually located within the gastric serosa. Recent experiments have shown that PMCs undergo MMT and actively promote gastric cancer infiltration. Therefore, it is necessary to reconsider whether preoperative peritoneal lavage should be used for gastric cancer staging as a standard operation. In addition, clinical reports of the recurrence of intraperitoneal implants after curative resection of early gastric cancer suggest that it is also necessary to enhance the evaluation of intraperitoneal implantation metastasis in patients with early-stage gastric cancer [110].

## 7. PMCs’ Transition Promotes Tumor Progression in the Peritoneal Cavity

Cancer-associated fibroblasts (CAFs), which are highly heterogeneous [111,112,113,114,115], are the main components of various tumor-associated microenvironments and are associated with a poor patient prognosis. CAFs promote tumor progression by releasing various chemokines and growth factors, which degrade the tumor extracellular matrix and induce cell activation [112,116,117]. CAFs originate from various precursor cells, including resident fibroblasts, peritumoral adipocytes, epithelial cells, hematopoietic hepatocytes, and endothelial cells [112,116,118,119,120]. CAFs do not have precise molecular markers as they differ among tumors [121].

Adult PMCs are characterized by differentiation into the endoderm, and are considered to be mesoderm-originating cells [122] that can differentiate into myofibroblasts, smooth muscle cells, endothelial cells, osteoblasts, and adipocytes [17,18,123,124,125]. Some studies have found that WT1-positive PMCs can be transdifferentiated into CAFs under a gastric cancer microenvironment using cell lineage-tracing technology [97]. In contrast, other studies utilizing cell lineage-tracing techniques provide a different perspective. WT1-positive PMCs were found to gradually accumulate on the peritoneal surface in a mouse model of peritoneal fibrosis induced by sodium hypochlorite, high-glucose dialysis solution, or TGF-β1, whereas α-SMA-positive myofibroblasts were mainly derived from PDGF+ fibroblasts that produce collagen I in the sub-peritoneal region [126]. WT1-positive PMCs only exist on the surface of the peritoneal cavity in postnatal and adult mice and cannot migrate to the deep layers and differentiate into other cell types. However, epicardial WT1-positive mesothelial cells can maintain the epicardial renewal ability, and promoted neogenesis and coronary angiogenesis in adult mice, suggesting that the transdifferentiation of PMCs is organ-specific [127]. It should be noted that this study only tracked the differentiation fate of PMCs in the normal physiological condition and did not observe the differentiation fate of PMCs in the pathological condition; therefore, these results should be validated in different models.

There is emerging evidence supporting the transdifferentiation of PMCs. Immunohistochemical staining of peritoneal specimens from gastric cancer patients with malignant ascites revealed that clusters of CAFs co-expressed the epithelial marker cytokeratin. Cell function experiments have provided strong evidence that PMCs acquire the phenotype of CAFs and displayed upregulation of cytokeratin and downregulation of E-cadherin expression, and further analysis indicated that TGF-β1 was an essential factor in inducing the transdifferentiation of PMCs [128]. Exosomes derived from malignant ascites in patients with gastric cancer showed high concentrations of TGF-β1, which increased the growth of peritoneal disseminated tumors by reducing the inhibitory effects of PMCs on tumor cells [129]. Moreover, TGF-β1 has been shown to induce the differentiation of PMCs in several studies [130,131].

PMC morphology has been shown to gradually change from a flat single layer to a cubic, fusiform multilayer spanning the peritoneal surface to the depths of metastatic lesions in individuals with colorectal peritoneal metastases. The PMCs markers CK7, calretinin, podoplanin, and WT1 are highly expressed on the peritoneal surface and are gradually less prevalent in the deeper layers. The expression levels of the mesenchymal marker desmin and the CAFs markers α-SMA and FAP gradually increase from the superficial to the deep layers. These patterns suggest that PMCs transdifferentiate into CAFs and migrate to deeper layers [69].

PMCs isolated from intraperitoneal metastases of ovarian cancer patients with ascites displayed a fibroblast-like morphology and co-expressed calretinin and α-SMA. When mice were co-inoculated subcutaneously with the ovarian cancer cell line SKOV3, the tumor volume significantly increased compared with that of mice inoculated with SKOV3 cells alone. Further experiments confirmed that the transdifferentiation of PMCs into CAFs was induced by TGF-β1/pSMAD3 [132].

The phenomenon of the transdifferentiation of PMCs was also identified in digestive and gynecologic tumors. Immunohistochemical results of intraperitoneal implant metastases in five human cases of ovarian cancer, four cases of colorectal cancer, three cases of pancreatic cancer, and one case of endometrial cancer were suggestive of the transdifferentiation of PMCs. The α-SMA-positive CAFs in the tumor stroma were observed to co-express the PMC marker calretinin in all tissue specimens examined, suggesting that some CAFs may be derived from transdifferentiated PMCs. The same phenomenon was echoed in animal experiments [133].

Although numerous studies have suggested the transdifferentiation of PMCs into CAFs (Figure 1D), this evidence has been chiefly obtained indirectly via observations of the co-expression of markers of PMCs and CAFs by immunohistochemical staining, and the conclusions derived from studies utilizing cell lineage-tracing technology are contradictory. These studies do not support mainstream theories regarding the transdifferentiation of PMCs. Moreover, they are primarily derived from physiological models or models of peritoneal fibrosis and lack high-grade tumor environment-tracing evidence. Therefore, further research into the tumor microenvironment is needed. 

## 8. Conclusions

Whether PMCs promote or inhibit peritoneal metastases remains controversial. It was previously believed that PMCs are the first barrier to be overcome for the dissemination of tumors in the abdominal cavity, suggesting that the presence of PMCs can prevent the further dissemination of tumor cells. However, more recent evidence suggests that PMCs can also promote tumor progression via their morphological changes under the persistent effects of the tumor microenvironment. Peritoneal cavity tumors can either induce the senescence of PMCs to generate SASP and promote local progression, or PMCs can undergo tumor-induced apoptosis to expose the connective tissue beneath the PMC layer for cell adhesion. Moreover, PMCs undergo MMT or transdifferentiate into CAFs under stimulation of tumor-soluble factors or extracellular vesicles, thereby forming a microenvironment that is conducive to tumor metastasis or actively promotes local tumor progression and peritoneal dissemination. The effect of PMCs on tumors is dynamic and may differ according to the stage of tumor infiltration or the pathological type.

This review highlights that the loss of the epithelial phenotype in PMCs is not the result of a single mechanism but is more likely a result of the synergy of multiple mechanisms. Elucidating the details of the interaction between PMCs and tumors to promote local progression and peritoneal dissemination requires further study. Maintaining the stability of the epithelial phenotype of PMCs may be an effective target for inhibiting peritoneal tumor dissemination and local progression.

## Data Availability

Not applicable.
